# A Case of Panophthalmitis Secondary to Odontogenic Maxillary Sinusitis

**DOI:** 10.7759/cureus.30801

**Published:** 2022-10-28

**Authors:** Garrett Mamikunian, Andrea Ziegler, Eric Thorpe

**Affiliations:** 1 Chicago Medical School, Rosalind Franklin University of Medicine and Science, North Chicago, USA; 2 Department of Otolaryngology – Head & Neck Surgery, Loyola University Medical Center, Maywood, USA

**Keywords:** streptococcus milleri, candida albicans, endogenous endophthalmitis, panophthalmitis, odontogenic maxillary sinusitis

## Abstract

Endogenous endophthalmitis is a bacterial or fungal infection within the eye that includes the vitreous and aqueous humors. Panophthalmitis is a subtype of endogenous endophthalmitis that also includes infection of the adjacent soft tissue of the orbit. We present a case of a 91-year-old female who initially presented with left eye pain and decreased vision. She was found to have panophthalmitis secondary to odontogenic maxillary sinusitis. To our knowledge, there has not been a case reported in the literature before that has established this association between panophthalmitis and odontogenic maxillary sinusitis.

## Introduction

Endophthalmitis is defined as a bacterial or fungal infection within the eye that also includes the vitreous and aqueous humors [1]. It is classified as either exogenous or endogenous. If it occurs exogenously, it typically occurs post-operatively or after trauma to the eye. Endogenous endophthalmitis (EE) occurs in the context of bacteremia and is further classified as focal, diffuse, or panophthalmitis [2]. Panophthalmitis is a type of EE that involves infection of the adjacent soft tissue of the orbit [3].

It has been shown that for patients with periodontal disease, there is a two-fold increase in maxillary sinus disease [4]. A disease arising from dental structures and causing maxillary sinusitis is known as odontogenic maxillary sinusitis (OMS) [5]. OMS has not previously been linked to panophthalmitis in the literature. To our knowledge, this is the first case reported in the literature of panophthalmitis resulting from an odontogenic left maxillary sinus infection that spread through an orbital floor defect of the left eye.

## Case presentation

A 91-year-old female with a history of atrial fibrillation, hypertension, hypothyroidism, coronary artery disease, and giant cell arteritis presented with left eye pain and decreased left eye vision that began three days prior to arrival. During these three days, she had pustular discharge from her left eye and was diagnosed with bacterial conjunctivitis by her primary care physician and given polymyxin B and trimethoprim. She then presented to the emergency department with weakness, dizziness, and chills and was found to have a urinary tract infection (UTI) secondary to Klebsiella pneumoniae for which she was started on ceftriaxone. A physical exam at the time revealed the patient’s left eye to be dilated and fixed with scleral erythema. There was mild proptosis and restricted extraocular movements in all directions. The patient was evaluated by ophthalmology and was diagnosed with EE. The patient was then started on vancomycin, moxifloxacin, and meropenem.

**Figure 1:**
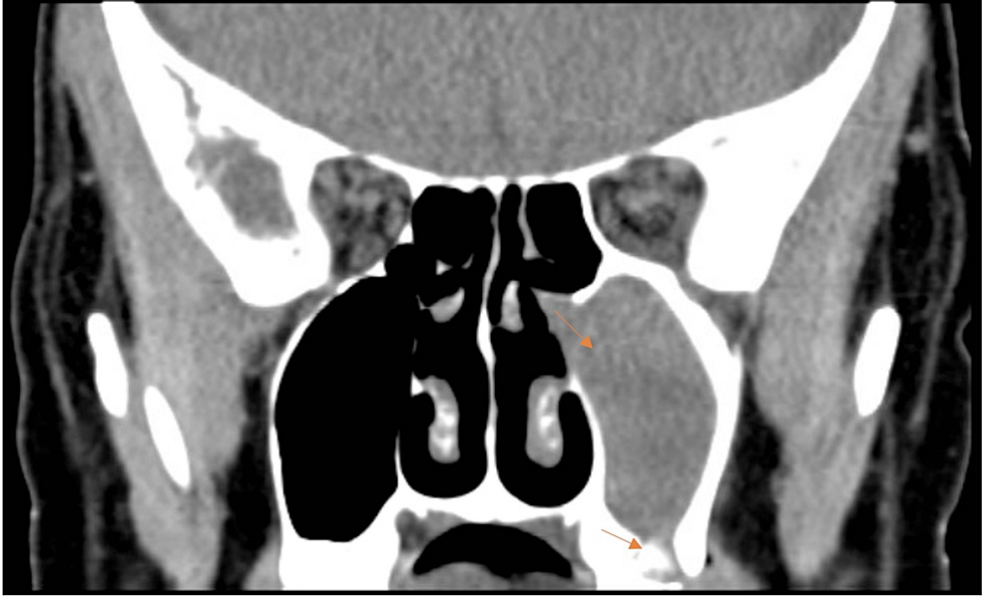
Coronal CT orbits image showing left maxillary second molar periapical lucency and left maxillary sinus opacification.

**Figure 2:**
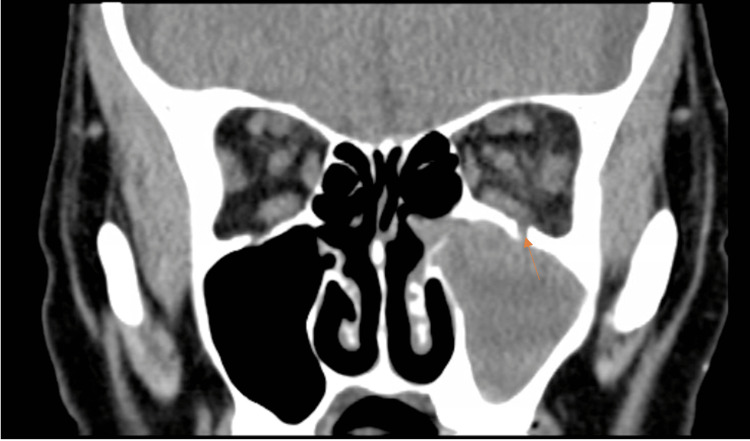
Coronal CT orbits image showing left maxillary sinus opacification and orbital floor defect.

**Figure 3:**
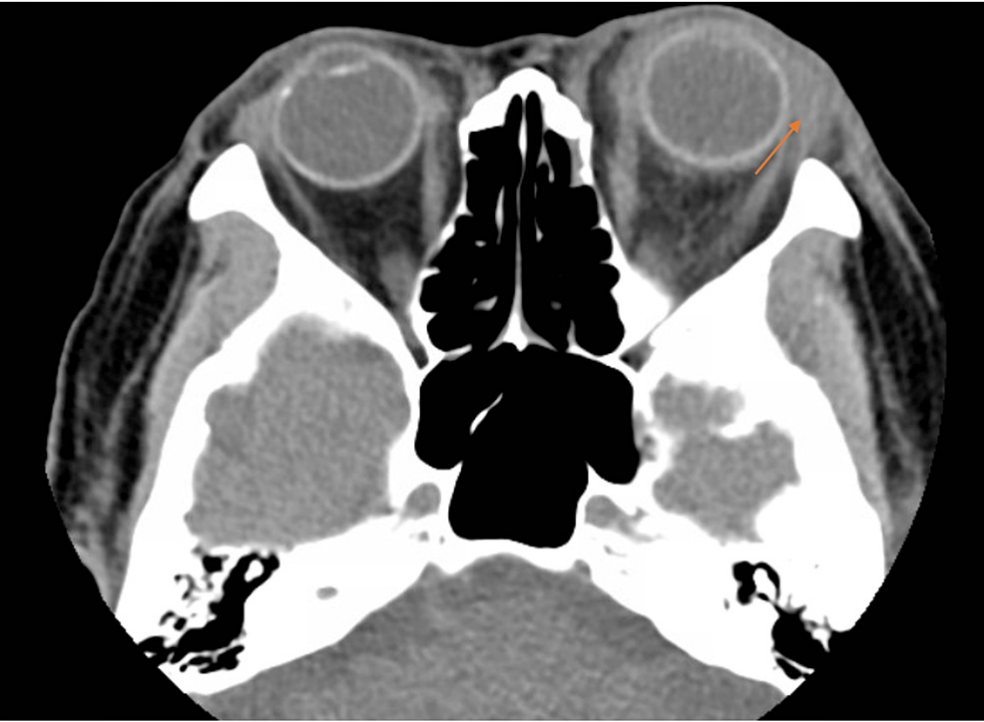
Axial CT orbits showing left proptosis with significant preseptal and postseptal edema.

On imaging, the patient was found to have a periapical abscess on tooth #14. A computed tomography scan of the orbits showed evidence of an odontogenic left maxillary sinus infection (Figure 1) along with an orbital floor defect of the left eye (Figure 2). Additionally, there was left proptosis with significant preseptal and postseptal edema (Figure 3).

It was believed that her local and regional infection was likely caused by odontogenic sinusitis. Oral and maxillofacial surgeons performed an extraction of tooth #14, while our team performed a left maxillary sinus Caldwell-Luc procedure in order to treat the source of the infection. Cultures from the maxillary sinus grew Candida albicans and Streptococcus milleri species. The infectious disease team started the patient on voriconazole. 

To rule out endocarditis as a possible source of infection, a transesophageal echocardiogram (TEE) was performed which was negative. Initially, due to the presence of the UTI, ophthalmology diagnosed the patient with EE; however, it was suspected that the patient’s left orbital floor defect allowed entry of the odontogenic left maxillary sinus infection causing opthalmology to eventually diagnose the patient with panophthalmitis of the left eye. The remaining hospital course was primarily uneventful; unfortunately, she did not regain the vision in her left eye.

## Discussion

To our knowledge, this is the first case of panophthalmitis in the literature that resulted from OMS. EE is a rare entity and only accounts for 2.6% of cases of endophthalmitis compared to exogenous which accounts for 97.4% of cases. Endophthalmitis is most commonly caused by bacteria (85.8%), trailed by fungi (14.2%) [6]. Maxillary sinus culture from our patient grew streptococcal species which follows previous findings that streptococcal species were the most common group isolated in EE [7]. Sinus cultures also grew Candida albicans, which is the most common cause of endogenous fungal endophthalmitis [8].

The diagnosis of EE is often confirmed through vitreous aspiration or vitrectomy. Vitrectomy has been shown to have a higher diagnostic yield compared to vitreous aspiration when diagnosing EE [9]. Differential diagnoses for EE can include herpes zoster ophthalmicus, sarcoidosis, Behcet syndrome, exogenous endophthalmitis, foreign body, and disseminated intravascular coagulation [10]. For candida EE, the treatment of choice is intravitreal amphotericin or voriconazole. Bacterial endophthalmitis is treated empirically typically with intravitreal vancomycin, cefazolin, or ceftazidime. Targeted therapy is started once culture results return [10].

It has been found that endocarditis and gastrointestinal (GI) tract infections were the most common source of EE [7]. Our patient received a TEE which was negative and she had no evidence of GI infection. With the patient’s history of UTI, it was initially thought that the patient had EE, likely due to bacteremia from her UTI. However, further workup showed OMS and an orbital floor defect suggesting direct extension into the orbit producing panophthalmitis.

The prognosis for regaining vision after EE is poor [11]. Unfortunately, the patient did not regain vision.

Our patient developed panophthalmitis in only her left eye which follows the literature as it typically occurs unilaterally [2]. It is unclear in the literature which eye is more likely to be involved in EE as it has been suggested that the right eye is most likely to be involved [12], while other studies have shown that the left eye is more likely to be affected [13]. The right eye is theorized to be more involved due to the right carotid artery having a more proximal and direct flow to the right eye [12].

A previous study showed the most common underlying disease of EE was diabetes mellitus and liver cirrhosis [14], neither of which our patient had. Additionally, EE has been found to have an association with pyogenic liver abscesses [15] and immunosuppression [10], which was also not found in our patient.

## Conclusions

This is a case of a 91-year-old woman who developed panophthalmitis and lost vision in her left eye secondary to OMS. This case study serves to make clinicians aware of the association between OMS and panophthalmitis, a subtype of EE. The development of panophthalmitis can place a patient’s vision at risk. Understanding this possible sequela of OMS will allow physicians to provide safer care to patients with OMS. To our knowledge, this is the first case in the literature that has established this association.
